# Paternal Resistance Training Modulates Calcaneal Tendon Proteome in the Offspring Exposed to High-Fat Diet

**DOI:** 10.3389/fcell.2020.00380

**Published:** 2020-06-16

**Authors:** Ivo Vieira de Sousa Neto, Ramires Alsamir Tibana, Leonardo Gomes de Oliveira da Silva, Eliene Martins de Lira, Gleyce Pires Gonçalves do Prado, Jeeser Alves de Almeida, Octavio Luiz Franco, João Luiz Quaglioti Durigan, Adetola B. Adesida, Marcelo Valle de Sousa, Carlos André Ornelas Ricart, Hylane Luiz Damascena, Mariana S. Castro, Wagner Fontes, Jonato Prestes, Rita de Cassia Marqueti

**Affiliations:** ^1^Laboratory of Molecular Analysis, Graduate Program of Sciences and Technology of Health, Universidade de Brasília, Distrito Federal, Brazil; ^2^Graduate Program of Physical Education, Universidade Católica de Brasília, Distrito Federal, Brazil; ^3^Graduate Program in Health Sciences, Universidade Federal do Mato Grosso, Cuiabá, Brazil; ^4^Graduate Program in Health and Development in the Midwest Region, Faculty of Medicine, Universidade Federal do Mato Grosso do Sul, Campo Grande, Brazil; ^5^Research in Exercise and Nutrition in Health and Sports Performance-PENSARE, Graduate Program in Movement Sciences, Universidade Federal do Mato Grosso do Sul, Campo Grande, Brazil; ^6^Center for Proteomic and Biochemical Analyses, Graduate Program in Genomic Sciences and Biotechnology, Universidade Católicade Brasília, Distrito Federal, Brazil; ^7^S-Inova Biotech, Graduate Program in Biotechnology, Universidade Católica Dom Bosco, Campo Grande, Brazil; ^8^University of Alberta, Divisions of Orthopaedic Surgery and Surgical Research, Edmonton, AB, Canada; ^9^Laboratory of Protein Chemistry and Biochemistry, Department of Cell Biology, Institute of Biological Sciences, Universidade de Brasília, Distrito Federal, Brazil

**Keywords:** overweight, intergenerational, paternal programming, exercise, tendon proteome

## Abstract

The increase in high-energy dietary intakes is a well-known risk factor for many diseases, and can also negatively impact the tendon. Ancestral lifestyle can mitigate the metabolic harmful effects of offspring exposed to high-fat diet (HF). However, the influence of paternal exercise on molecular pathways associated to offspring tendon remodeling remains to be determined. We investigated the effects of 8 weeks of paternal resistance training (RT) on offspring tendon proteome exposed to standard diet or HF diet. Wistar rats were randomly divided into two groups: sedentary fathers and trained fathers (8 weeks, three times per week, with 8–12 dynamic movements per climb in a stair climbing apparatus). The offspring were obtained by mating with sedentary females. Upon weaning, male offspring were divided into four groups (five animals per group): offspring from sedentary fathers were exposed either to control diet (SFO-C), or to high-fat diet (SFO-HF); offspring from trained fathers were exposed to control diet (TFO-C) or to a high-fat diet (TFO-HF). The Nano-LC-MS/MS analysis revealed 383 regulated proteins among offspring groups. HF diet induced a decrease of abundance in tendon proteins related to extracellular matrix organization, transport, immune response and translation. On the other hand, the changes in the offspring tendon proteome in response to paternal RT were more pronounced when the offspring were exposed to HF diet, resulting in positive regulation of proteins essential for the maintenance of tendon integrity. Most of the modulated proteins are associated to biological pathways related to tendon protection and damage recovery, such as extracellular matrix organization and transport. The present study demonstrated that the father’s lifestyle could be crucial for tendon homeostasis in the first generation. Our results provide important insights into the molecular mechanisms involved in paternal intergenerational effects and potential protective outcomes of paternal RT.

## Introduction

The global obesity disease is often causally linked to marked changes in lifestyle and diet, such as increases in high-energy dietary intakes and low levels of physical activity (Hruby and Hu, 2015). In animal models, consumption of high-fat (HF) diet promotes deleterious effects on morphological, biomechanical and biochemical properties of tendons (Biancalana et al., 2010; Boivin et al., 2013). Previous data have shown that rats exposed to HF diet displayed an increase of oxidized low-density lipoprotein deposition in extracellular matrix (ECM) as well a reduction of failure stress in the patellar tendon when compared with rats exposed to a standard diet (Grewal et al., 2014). Zucker rats displayed disorganized collagen fibril bundles and decreased average fibril diameter accompanied by adverse effects in displacement at maximum load and maximum strain, indicating a significant harmful ECM remodeling in deep digital flexor tendon (Biancalana et al., 2010). In this way, further investigations are necessary to determine strategies to attenuate these detrimental responses on tendons.

Ancestral environmental conditions can elicit lifelong effects on the offspring physiology, including increased susceptibility to the obesity and metabolic disorders development (Krout et al., 2018; Winther et al., 2018; Glendining and Jasoni, 2019). Although detrimental effects of maternal obesity during perinatal periods on adiposity and metabolic function in offspring are well established, paternal lifestyle may also influence offspring developmental outcomes. It has been reported that predisposing to obesity in adulthood for children increases around 83% if only one parent (father or mother) are obese (Whitaker et al., 1997; Dorosty et al., 2000). Paternal obesity is a risk factor for increased adiposity and glucose intolerance in the adult offspring (Ng et al., 2010; McPherson et al., 2015), besides harming the sperm metabolic function and fertility (McPherson et al., 2015). Therefore, paternal intergenerational inheritance has important implications in the offspring phenotype.

The protection of future unborn generations must be a paramount consideration, because it might contribute to a range of implications for offspring health. A recent study indicates that voluntary paternal exercise (running wheel) suppresses the detrimental effects of paternal HF diet on offspring, reducing the harmful effects on glucose tolerance, fat mass and glucose uptake in different skeletal muscles (Stanford et al., 2018). Krout et al. (2018) demonstrate that paternal exercise (2 weeks of wheel running) increases the expression of insulin signaling pathways in offspring skeletal muscle, what can be an important tool to prevention type 2 diabetes. Although highly significant, the authors did not strictly manipulate training variables (duration, intensity, rest interval, training volume), which could amplify the understanding of exercise dose on metabolic variables, and tendon offspring. Considering the intimate relationship between the skeletal muscle and tendon unit, it might be reasonable that controlled paternal exercise may modulate the molecular pathways related to offspring tendon remodeling.

DNA, histone methylation, and small RNAs are the most thoroughly studied potential intergenerational mechanisms (Stanford et al., 2018), while other molecular processes can be relevant. This variety of modifications can change chromatin structure or recruit transcriptional cofactors to DNA in order to regulate gene expression and genome activity, which consequently might alter protein profile in a cell. Proteins are effectors of cellular mechanisms, and are critical players in the maintenance of cellular homeostasis (Malipatil et al., 2019). Proteomic analysis and bioinformatics tools allow an integrated view of the molecular pathways modulated by diet and exercise training (Kleinert et al., 2018). In a recent study, we examined the effects of resistance training (RT) on the protein profile of calcaneal tendon during aging. Barin et al. (2017) demonstrated that RT (12 weeks, three times per week) up-regulates protein abundance levels related to ECM organization, the stability of the ciliary architecture and metabolic processes in old trained rats when compared with old sedentary rats. The authors demonstrated the relevance of RT as an intervention to attenuate the detrimental effects inherent to aging-associated changes (Barin et al., 2017). Relevantly, exercise programs can be often more successful than pharmacological interventions to protect against tendon weakening processes (Snedeker and Foolen, 2017). RT is considered an important non-pharmacologic agent capable of inducing significant effects on structural and mechanical properties and collagen synthesis in tendons (Marqueti et al., 2018), while other training modalities may have limited effects on EMC tendon remodeling (Buchanan and Marsh, 2001; Boivin et al., 2013).

The identification and quantification of key proteins from different organelles, biologic process, and molecular functions would provide new insights into the molecular pathways involved in paternal intergenerational inheritance. A mechanistic understanding of these effects can provide crucial clues for the development of therapeutic approaches to mitigate the tendon disorders associated with HF diet. The purpose of this study was to investigate the effects of 8 weeks of paternal RT in offspring tendon proteome exposed to control and HF diet. We found evidence supporting the hypothesis that paternal RT regulates protein abundance levels directly related to the maintenance, organization, and integrity of the tendon ECM and that such regulation is enhanced by different diets.

## Materials and Methods

### Animals and Grouping

The proper care and use of laboratory animals in research were conducted following the Guide [National Research Council, 2011 (Council, 2010)]. The research protocol received approval from the Ethics Committee on Animal Experimentation from the Catholic University of Brasilia (Protocol No. 010/13).

First, 4-month-old male Wistar rats (*Rattus norvegicus albinus*, weighing ± 376 g) were placed in collective cages (3 or 2 rats per cage), and were randomly divided into two groups (five animals per group): sedentary fathers (SF; did not perform RT, free to move around the cages) and trained fathers (TF; performed RT). The offspring were obtained by mating with sedentary females. After the 8 weeks of paternal RT, the estrous cycle in female rats was verified daily, and during proestrus phase, one male and one female were housed together for two consecutive days for mating, during which they were allowed free access to a control diet (Purina^®^, Descalvado-SP, Brazil). The experimental groups in the current study were composed of 20 male pups. Litters were standardized among five pups each to avoid litters of different sizes, which were left together with their mothers until they were weaned. Litters belonging to the same experimental group were offspring of different parents.

**FIGURE 1:**
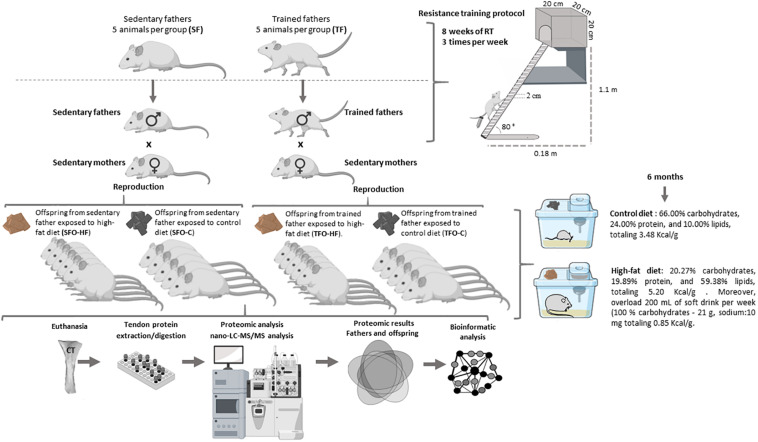
Experimental design. Schematic representation of the methodological sequence followed in the study.

Male offspring were weaned and divided into four groups (five animals per group): offspring from sedentary father exposed to either control diet (SFO-C) or to high-fat diet (SFO-HF), and offspring from trained father exposed to either control diet (TFO-C), or to high-fat diet (TFO-HF). All animals came from the Central Vivarium of the Faculty of Physical Education of the Catholic University of Brasilia. The animals were housed in polypropylene cages (maximum three rats per cage) at a temperature of 23 ± 2°C with 12:12 h dark: light cycle. The offspring were weighed and evaluated weekly for 6 months using a digital scale (Filizola^®^, São Paulo, Brazil). Study experimental design is presented in Figure 1. Not including female offspring in present study has been justified due to the variable nature of female data caused by hormonal fluctuations associated with the female reproductive cycle.

### Offspring Diet

The offspring groups exposed to control diets (SFO-C and TFO-C) were fed with standard feed (66.00% carbohydrates, 24.00% protein, and 10.00% lipids, totaling 3.48 Kcal/g, Labina Presence^®^, Paulinia, São Paulo, Brazil) and water *ad libitum*. Females were also kept on the same control diet throughout gestation and lactation. The SFO-HF and TFO-HF groups were exposed to HF diets commercially purchased (20.27% carbohydrates, 19.89% protein, and 59.38% lipids, totaling 5.20 Kcal/g) (Prag solutions^®^, Biosciences, Jau, Brazil) and overload of 200 mL of soft drink per week (100% carbohydrates - 21 g, sodium:10 mg totaling 0.85 Kcal/g), and with free access to water after the 21st day of birth, during 6 months. Previous studies showed the efficacy of this HF diet on body weight gain, adipose tissue weight gain in Wistar rats (Thalita et al., 2014; Goncalves et al., 2018). The use o soft drinks for rats is a complement and characterization of the HF diet, besides it’s an effective strategy for an increase the total energy intake and body mass over time. Soft drink industrialized is an element to consider for the development of an *in vivo* model of obesity that is most representative of the pathophysiology of the disease in humans (Alkhedaide et al., 2016). The offspring food was weighed weekly using a digital scale (Filizola, São Paulo, Brazil) and food intake (amount offered – the amount remaining in the cage) was monitored.

### Paternal Resistance Training

The RT protocol was modified from Hornberger and Farrar (2004), according to the requirements of our study. Training procedures were also described elsewhere (de Sousa Neto et al., 2017, 2018; Tibana et al., 2017; Marqueti et al., 2018). During the 8 weeks of RT, climbing sessions were performed three times per week (Monday, Wednesday, and Friday) during the afternoon (between 2:00 and 4:00 p.m.). Before the treatment period, an RT adaptation protocol was managed, which required the animals to climb a vertical ladder (1.1 m × 0.18 m, 2-cm grid, 80° incline) with weights attached to their tails. The animals performed 8–12 dynamic movements per climb. The load apparatus was fixed to the tail by wrapping the proximal portion of the tail with an adhesive tape. The rats were placed at the bottom of the ladder and familiarized with climbing. If necessary, a stimulus was applied, with tweezers, to the animal’s tail to initiate the movement. At the top of the ladder, the rats reached a housing chamber (20 × 20 × 20 cm), where they were rested for 2 min. This procedure was repeated until the rats would voluntarily climb the ladder for three consecutive turns without stimulus. The animals performed three sections of familiarization with a 48 h interval.

The first training session started 3 days after the familiarization period and consisted of 4–8 ladder climbs with progressively heavier loads. The initial climb consisted of a load that was 75% of the animal’s body mass. Upon successful completion of this load, an additional 30 g weight was added until the rat could not climb the entire ladder with such load. Failure was determined when the animal could not progress up the ladder after three successive stimuli to the tail. The highest load successfully carried the entire length of the ladder was considered to be the rat’s maximal carrying capacity for that training session. After determining the maximum load capacity, training sessions for the FT group consisted of eight ladder climbs with two sets of each load 50, 75, 90, and 100% of their maximal carrying capacity interspersed by 120 s of intervals between each set. Previous studies demonstrated beneficial effects of this RT protocol on molecular, morphologic/structural, biochemical properties and function of CT (Barin et al., 2017; Marqueti et al., 2018).

### Treadmill Running Test

Treadmill running was used for assessing the maximal velocity in offspring and was completed in the last 2 weeks of diet exposure according to the protocol established by Rosa et al. (2016). Procedures are described elsewhere (Copp et al., 2009; Almeida et al., 2013). The test began with treadmill running familiarization in order to minimize the stress related to exercise. The rats were initially familiarized with running on a treadmill designed for small animals (li 870, Letica Scientifi, Barcelona, Spain) during 3 days with a constant speed of 13 m.min^–1^ during 10 min. After this, the test protocol started 2 days after the familiarization. During the incremental protocol, the rats started running at a speed of 13 m.min^–1^, followed by speed increments of 3 m.min^–1^ every 3 min until they reached fatigue. The fatigue was defined as the point at which the animals were no longer able to maintain their pace with the treadmill, even when exposed to light electrical stimulation. The maximal aerobic velocity was established by the antecedent stage as proposed by Almeida et al. (2013).

### Metabolites Analysis

After an expose of 24 weeks to diet, serum glucose, triglycerides, and cholesterol were measured using a glucometer (ACCU CHECK- Active, Roche, Mannheim, Germany), and their respective reagent tapes, according to the manufacturer’s recommendations. Data related to Metabolites is presented in mg/dl.

### Euthanasia

In order to avoid the acute effects of RT, the fathers were euthanized 48 h after the end of the training period with an intraperitoneal injection of xylazine solution (12 mg/kg of body weight) and ketamine (95 mg/kg of body weight). The offspring were euthanized using the same combination of solutions after 24 weeks of exposure to diet. Chronic tendon disorders, most commonly affecting the calcaneus tendon (CT), represents approximately 50% of all sports injuries (Barin et al., 2019). Moreover, CT has a relevant biomechanical function in response to RT, which justifies the choice of use CT (Guzzoni et al., 2018). The CT was immediately dissected from the posterior right paw and frozen in clean microtubes using liquid nitrogen and then stored at −80°C. All tendons were dissected by an experienced researcher in order to prevent contamination of other tissues. The sample preparation methodology, including protein extraction, protein digestion, chromatography and nano-LC-MS/MS analysis, database search and label-free quantification was adapted from Cury et al. (2019).

### Tissue Extraction

The entire tendon (approximately 80 mg) from fathers and offspring was grounded in liquid nitrogen using mortar and pestle. Then, the sample was added to a solution of 10% (w/v) trichloroacetic acid and 0.07% (v/v) β-mercaptoethanol in cold acetone; the resulting suspension was thoroughly mixed by vortexing, and thereupon incubated for 3 h at 4°C. After incubation, samples were centrifuged at 10,000 *g* for 20 min at 4°C. Next, the supernatant was removed, and the remaining pellet was washed five times with 10% (w/v) trichloroacetic acid in acetone until the complete disappearance of pigments. The pellet was dried using a Speed-Vac concentrator and resuspended in rehydration buffer (7 M urea, 2 M thiourea, 250 mM TEAB, pH 8.5). Protein concentration was determined by using Qubit^®^ 2.0 assay (Invitrogen, Carlsbad, CA, United States) and extracted protein quality was assessed in 10% SDS-PAGE.

### Protein Digestion

The proteomics analysis experiments were conducted by a blinded researcher, attenuating possible bias related to this process. The extracted proteins (200 μg) were reduced with 10 mM dithiothreitol (DTT) for 60 min at 56°C and alkylated with 100 mM iodoacetamide (IAA) for 60 min at 37°C in the dark. Then, the samples were diluted in 100 mM ammonium bicarbonate, pH 8.1. Subsequently, the alkylated proteins were digested with trypsin (1:50 v/v – Promega, Madison, WI, United States) at 37°C for 16 h. After digestion, the resulting peptide solution was acidified with 0.1% trifluoroacetic acid and centrifuged at 10,000 *g* for 10 min. Next, the supernatant was desalted in homemade microcolumns (C18 stage tips), vacuum-dried, and resuspended in 0.1% (v/v) formic acid. The sample was quantified using Qubit^®^ 2.0 (Invitrogen, Carlsbad, CA, United States) for further analysis by liquid nano-chromatography coupled to a mass spectrometer.

### Nano-LC-MS/MS Analysis

The chromatography and mass spectrometry analysis was performed according to Cury et al. (2019) and adapted for proteomic assay in the intergenerational study. The tryptic peptides were applied to a Dionex Ultimate 3000 liquid chromatographer (Sunnyvale, United States) for reversed phase nano-chromatography as following: three technical replicates of 1 μg from each biological replicate were injected into a trap column (2 cm × 100 μm), containing C18 5 μm particles. The samples were eluted to an analytical column (32 cm × 75 μm, C18 3 μm), and then to the ionization source of the spectrometer. The elution gradient was composed of 0.1% (v/v) formic acid in water (solvent A), and 0.1% (v/v) formic acid in acetonitrile (solvent B), in a gradient of 2–35% solvent B for 180 min. The eluted fractions were directly sprayed in the ionization source of an LTQ Orbitrap Elite mass spectrometer (Thermo Fisher Scientific, Germany) and analyzed in DDA (data-dependent acquisition) mode. The MS1 spectra were obtained in the Orbitrap analyzer (resolution of 120,000 FWHM at 400 m/z) in the range of 300–1650 m/z. For each MS1 spectrum, the 15 most intense ions were automatically chosen and directed to high-energy collision-induced dissociation (HCD) fragmentation. Repeated fragmentation of the same precursor was prevented by dynamic exclusion for 90 s. The configuration for HCD was: insulation window 2.0 m/z, automatic gain control (AGC) of 5 × 10^6^, collision energy normalized to 35% and the threshold for the selection of 3000.

### Database Search and Label-Free Quantification

Methods regarding database and label-free quantification were based on previous studies from our research group (Barin et al., 2017; Cury et al., 2019). Initially, the files obtained from the mass spectrometer were analyzed using the software Progenesis IQ for alignment of the MS1 peaks found in the chromatograms, extracted ion chromatogram (XIC)-based quantification and normalization. After the peptide peaks were quantified and grouped, the identification of proteins was performed using Peaks 7.0 software, which deduced sequences from the fragmentation information. The database used in this study included the UniProt, SwissProt (Switzerland, Geneva), and searches focused on the *Rattus* spp. taxonomy. The search parameters used were: precursor ion mass error tolerance of 10 ppm, MS/MS mass tolerance of 0.05 Da, carbamidomethylation of cysteine residues (fixed modification) and deamidation and methionine oxidation (variable modifications). Trypsin was selected as the digestion enzyme, and up to two missed cleavage sites per peptide were allowed. The extracted peak area of each MS1 ion was calculated and these values were taken into consideration for the intensity calculations and subsequent comparison with the other groups. The identified proteins were filtered at 1% for false discovery rate (FDR), and a minimum of 1 unique peptide per protein was required for identification. The protein identification information was imported into the Progenesis IQ software, which combined them with previously generated quantitative data. For a qualitative analysis, the spectra were submitted to the Peaks software using the same parameters, but this search was not restricted to regulated events as in the quantitative analysis. The inclusion criterion for identified proteins in the qualitative analysis was the presence in at least four of five animals from each group, increasing rigor.

### Generation of PPI Network

The proteins identified as differentially abundant were investigated for their interaction with other proteins using bioinformatics tools via STRING Interactome 10.0 (Search Tool for the Retrieval of Interacting Genes/Proteins), accessible at https://string-db.org (Szklarczyk et al., 2015). The STRING 10.0 analysis was performed using the medium confidence score (0.400) to compare our filtered data with homologous proteins of *Rattus norvegicus* database.

### Statistical Analysis

Proteins with up-regulation and down-regulation were expressed as histograms through distributions of abundance ratios. Initially, proteins were also filtered by delta (Δ) of at least (≥) 0.5-fold change, increasing rigor. Shapiro –Wilk and Levene’s tests were used to analyze the homogeneity of the variance. Independent t-test was used for comparison of protein abundance levels between father’s groups. A two-way independent ANOVA (training and diet as factors) was used to compare protein abundance levels between offspring groups. When a significant difference was detected, a Tukey *post hoc* test was applied to identify the differences. An alpha threshold of 0.05 was considered for significance. The software GraphPad Prism 7.0 (San Diego, CA, United States) was used for statistical analysis and graphics design. Principal Components Analyses (PCA) and Heatmaps were prepared with Metaboanalyst software^1^. The power of the sample size for main proteins was verified by *post hoc* using G*Power version 3.1.9 (Kiel University, Kiel, Germany) (Supplementary Data 1).

## Results

### Effects of Diet and Paternal RT on Body Weight, Adiposity, Food Intake, Metabolites, and Treadmill Running Test

The growth trajectory and physiologic analysis of the offspring are shown in Table 1. During the period between the pre-diet week, and 24 weeks of diet administration a significant body weight gain was observed in the animals fed with HF-diet compared to the groups receiving a standard diet. There was no difference between TFO-C and SFO-C group, while paternal RT prevented the body weight gain, and reduced adiposity markers in the TFO-HF when compared to SFO-HF group after 24 weeks (*p* = 0.001).

**TABLE 1:** Growth trajectory of the offspring.

	SFO-C	TFO-C	SFO-HF	TFO-HF
**Body weight**				
Birth weight (g)	29.34 ± 1.46	27.83 ± 1.66	32.64 ± 1.47	31.92 ± 1.26
Weaning weight (g)	32.6 ± 1.62	30.92 ± 1.84	36.27 ± 1.64	35.4 ± 1.40
Final body weight (g)	331.36 ± 12.97	311.12 ± 22.81	433.9 ± 31.06^a,b^	398.84 ± 7.30^a,b,c^
**Adiposity markers**				
Total visceral adipose weight (mg)	4.26 ± 0.94	3.39 ± 1.34	13.46 ± 2.08^a,b^	6.91 ± 2.22^a,b.c^
Adiposity index (%)	1,29 ± 0.24	1,08 ± 0.36	4,75 ± 0.95	2,63 ± 0.68
Cross-Sectional Area of adipocyte (μm^2^)	3699.04 ± 548.27	3256.7 ± 306.59	5891.68 ± 839.35^a,b^	4473.6 ± 561.60^*c*^
**Food intake**				
Average caloric intake of feed (kcal)	459.51 ± 120.27	400.89 ± 103.75	551.74 ± 50.49^a,b^	546.63 ± 47.67^a,b^
Average caloric intake of soft drink (kcal)	20.4	20.4	20.4	20.4
Feed efficiency (%)	10.1%	11.10%	22.00%	19.90%
**Metabolites**				
Serum Glucose (mg/dl)	92.00 ± 9.79	93,33 ± 1.77	105.75 ± 16.48 ^a,b^	112.25 ± 11.86^a,b^
Serum Cholesterol (mg/dl)	156.50 ± 3.87	158.25 ± 4.79	168.40 ± 5.08^a,b^	163.00 ± 1.67
Serum Triglycerides (mg/dl)	132.0 ± 12.28	128.8 ± 3.96	142.0 ± 11.67	138.4 ± 18.24
**Functional test**				
Maximal velocity (m/min)	48.3 ± 3.16	45.3 ± 2.44	46.3 ± 7.48	49.3 ± 2.0
Maximal distance obtained in the test (m)	447.4 ± 38.45	429.0 ± 35.11	375.2 ± 84.41	407.8 ± 45,16

*Values are presented as means ± SD. SFO-C, offspring from sedentary fathers, exposed to control diet; TFO-C, offspring from trained fathers exposed to control diet; SFO-HF, offspring from sedentary fathers exposed to high-fat diet; TFO-HF, offspring from trained fathers exposed to a high-fat diet. Statistically significant differences compared to: ^*a*^SFO-C; ^*b*^TFO-C; ^*c*^SFO-HF, *p* ≤ 0.05 (*n* = 5 per group).*

The offspring exposed to HF-diet consumed fewer grams of food overall compared to the groups receiving a standard diet (*p* = 0.001), yet they presented higher overall caloric intake (kcal) (*p* = 0.001) and feed efficiency ratio (22.0% vs. 10.1%). There was no difference in food and calories intake between offspring from sedentary and trained fathers receiving different diets (*p* > 0.05). Furthermore, the SFO-HF and TFO-HF groups consumed the same amount of soft drink.

Serum glucose and cholesterol levels significantly increased in the SFO-HF group as compared with the SFO-C and TFO-C groups (*p* = 0.001 and *p* = 0.01, respectively), while there was no difference between SFO-HF and TFO-HF groups (*p* > 0.05). There was no difference between groups on serum triglycerides and maximal velocity in treadmill test.

### Functional Proteome Annotations

A qualitative overview of all identified proteins show 388 proteins identified by Nano- LC-MS/MS analysis (Supplementary Data 2), among them 384 (fathers) and 383 (offspring) proteins met the inclusion criterion and were classified according to UniProt database and Panther classification system in the biological process, molecular function and cellular localization.

Supplementary Figure 1A shows the identified proteins according to their cellular localization (A), biological process (B), and molecular function (C). In the present investigation, most of these proteins were derived from the cytoplasm (22.78%) followed by the nucleus (13.86%), cytoskeleton (12.62%), and extracellular matrix (9.66%). Additionally, the biological process classification showed that 20.09% of these proteins were related to metabolic processes followed by transport (9%), cytoskeleton organization and others (7.71%), (Supplementary Figure 1B). Finally, the primary molecular function observed was binding (34.95%), followed by enzymatic (24.68%), structural (15.58%), protease inhibitor (5.10%), and others (4.67%) (Supplementary Figure 1C).

Venn diagram was used to show the distribution of the identified proteins into two experimental groups (Supplementary Figure 2A). From the 384 proteins identified in the fathers’ samples, 376 were common to both groups, while 1 protein were identified only in the sedentary fathers’ group (SF) and 7 proteins were identified only in the trained fathers’ group (TF). The protein identified only in the SF group was mainly related to translation (Eukaryotic translation initiation factor 4A1). The proteins identified only in the TF were associated with cell component organization (Protein Synm and Thymosin beta-10), metabolic process (Isocitrate dehydrogenase [NADP]), proteolysis (Cathepsin D), myelinization (Myelin protein P0), biological regulation (Isoform Short of 14-3-3 protein beta/alpha) and transcription regulation (Protein Edrf1).

Regarding the four offspring groups, from the 383 identified proteins, 369 were common to all groups, while 3 proteins were identified only in the offspring from sedentary father exposed to high-fat diet (SFO-HF) and 1 protein was identified only in the offspring from trained father exposed to high-fat diet (TFO-HF). The high proportion of proteins identified in all groups confirms the consistency in the sample preparation. The proteins identified only in the SFO-HF group were mainly related to cell component organization (Keratin, type I cytoskeletal 17), myelinization (Myelin protein P0) and transcription regulation (High mobility group nucleosomal binding domain 2). The protein identified only in the TFO-HF was associated with the cell cycle (Protein S100) (Supplementary Figure 2B).

### Principal Components Analyses (PCA) and Heatmaps

**FIGURE 2:**
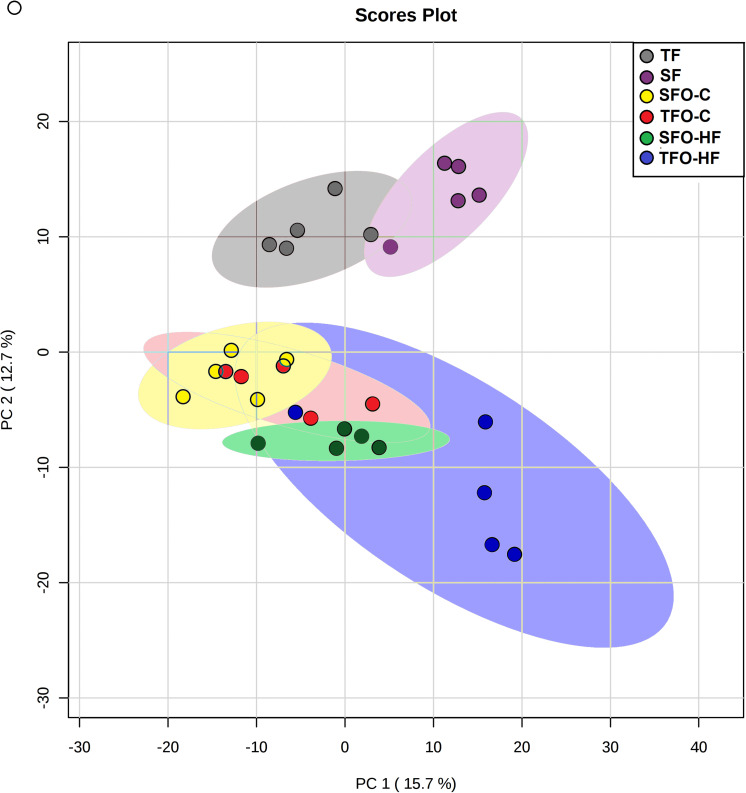
The results of Principal Components Analyses (PCA). Score plot of proteomic data set acquired by Nano-LC-MS/MS of the six biological groups: SF, sedentary fathers (gray); TF, trained fathers (purple); SFO-C, offspring from sedentary fathers, exposed to control diet (yellow); TFO-C, offspring from trained fathers exposed to control diet (red); SFO-HF, offspring from sedentary fathers exposed to high-fat diet (green); TFO-HF, offspring from trained fathers exposed to a high-fat diet (blue). Each animal is represented by individual points.

**FIGURE 3:**
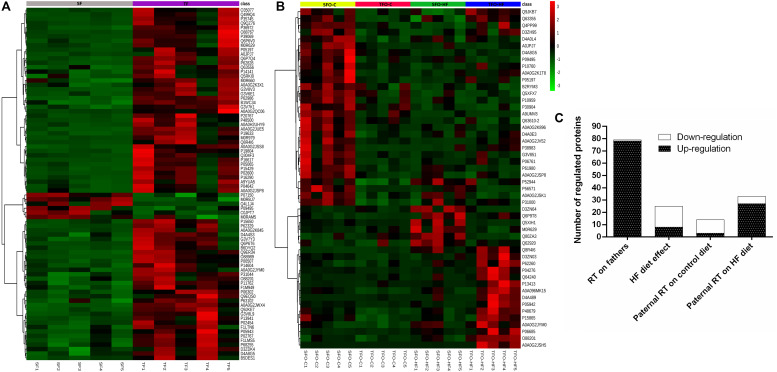
Heat map of the changes for each animals in the fathers **(A)** and offspring groups **(B)**. Each horizontal line represents an individual protein. The top 80 proteins regarding fathers and 50 from offspring, respectively. SF, sedentary fathers; TF, trained fathers; SFO-C, offspring from sedentary fathers, exposed to control diet; TFO-C, offspring from trained fathers exposed to control diet; SFO-HF, offspring from sedentary fathers exposed to high-fat diet; TFO-HF, offspring from trained fathers exposed to a high-fat diet. Read counts for each animal has been plotted as log2. Green and red indicate a decrease and increase of protein abundance levels, respectively. Overall number of regulated (down-regulation or up-regulation) protein for training and diet as factors **(C)**.

Results from the PCA in fathers and offspring are shown in Figure 2. The purpose of this analysis was to reveal patterns of protein abundance levels that may distinguish the experimental groups from one another. The 30 dots in the figure represent the five SF (gray), five TF (purple), five SFO-C (yellow), five TFO-C (red) five SFO-HF (green), and five TFO-HF (blue) samples. Notably, there is distinct clustering among the fathers groups, with the two sample groups occupying separable, non-overlapping, regions in the PCA plot. On the hand, overlapping was present among the SFO-C and TFO-C group. Finally, there were clear differences in the patterns of protein abundance levels among the others offspring groups (SFO-C, SFO-HF and TFO-HF), suggesting that paternal RT modulates the protein profile of the calcaneal tendon, especially noticeable in offspring exposed to HF diet. The variability in data captured by PCA along PC1 and PC2 were 15.7 and 12.7%, respectively (Figure 2). Figure 3 displays a heat map normalized abundance levels from each father and offspring. As expected, there were notable differences in the protein levels among fathers (Figure 3A). The SFO-C and TFO-HF groups showed majority high abundance levels when compared with TFO-C and SFO-HF groups, respectively (Figure 3B). Considering the criteria for down-regulation and up-regulation, the abundance of 79 proteins was shown to be altered in fathers after RT (78 protein upregulated and 1downregulated). The abundance of 25 proteins was shown to be altered by HF diet (8 protein upregulated and 17 downregulated). Paternal RT modified 14 proteins in offspring exposed to control diet (3 proteins upregulated and 11 downregulated) and 33 proteins in offspring exposed to HF diet (27 proteins upregulated and 6 downregulated), respectively (Figure 3C).

### Father’s Tendon Proteome

**FIGURE 4:**
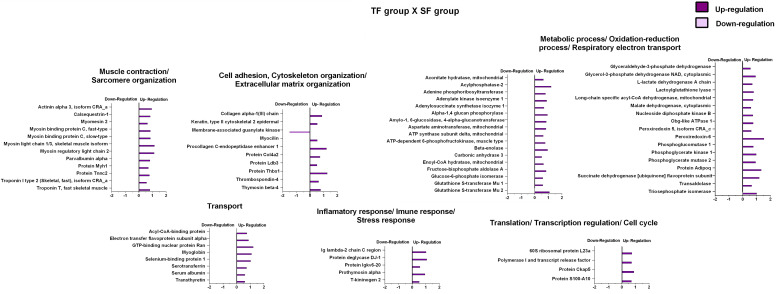
Effects of resistance training on father tendon proteome. Analysis of proteins from the tendons of the trained versus control animals (fathers). Histogram of protein abundance levels from intergroup analysis considering only proteins with down-regulation and up-regulation (*p* ≤ 0.05), with a ln(fold change) of at least (≥) 0.5. SF, sedentary fathers; TF, trained fathers. The *X*-axis represents the natural logarithm of the ratio between the treatments (purple: **TF:SF ratio**). All altered proteins are grouped according to their biologic process as noted in Gene Ontology (GO). The proteins were considered reliably identified only if presenting an FDR < 1% and at least two matching peptides. Supplementary Data 3 shows detailed information about each protein outlined in this figure.

The effects of resistance training on paternal tendon protein abundance levels were grouped according to their biological processes and are presented in Figure 4 and Table 2. Supplementary Data 3 shows fold-change, *p*-values *t*-test related to regulated proteins. From TF group, abundance levels of 79 proteins were shown to be altered as compared with the SF group (78 proteins increased and 1 decreased). In this analysis (TF:SF), proteins were mainly related to muscle contraction and sarcomere organization (12 upregulated), cell adhesion, cytoskeleton organization/extracellular matrix organization (9 upregulated and 1 downregulated); metabolic process, respiratory electron transport and oxidation-reduction process (34 upregulated), transport (8 upregulated), inflammatory response/immune response/stress response (5 upregulated), translation and transcription regulation, cell cycle (4 upregulated) and miscellaneous (6 upregulated).

**TABLE 2:** Protein abundance levels related to miscellaneous functions from TF:SF analysis considering only proteins with up-regulation and down-regulation (*p* ≤ 0.05) with a delta (Δ) of at least (≥) 0.5-fold change.

Protein description	Primary name	Biological process	*p*-value	Log (e)
				Fold change
TF:SF				
14-3-3 protein epsilon	P62260| 1433E_RAT	Biological regulation	0.0415	0.793116678
Bcl2-associated athanogene 3	Q5U2U8| Q5U2U8_RAT	Apoptotic process	0.0445	0.665601129
Elongation factor 2	P05197| EF2_RAT	Biosynthetic process	0.0148	0.952602494
Galectin-1	P11762| LEG1_RAT	Apoptotic process	0.0006	0.679780043
Protein Vps13d	A0A0G2JYD4| A0A0G2JYD4_RAT	Biological regulation	0.0391	1.310641532
Taste receptor type 2 member 41	Q9JKE7| T2R41_RAT	Response to stimulus	0.0014	0.881750158

### High-Fat Diet Cause a Reduction in the Abundance of Proteins Related to ECM Tendon Remodeling

**FIGURE 5:**
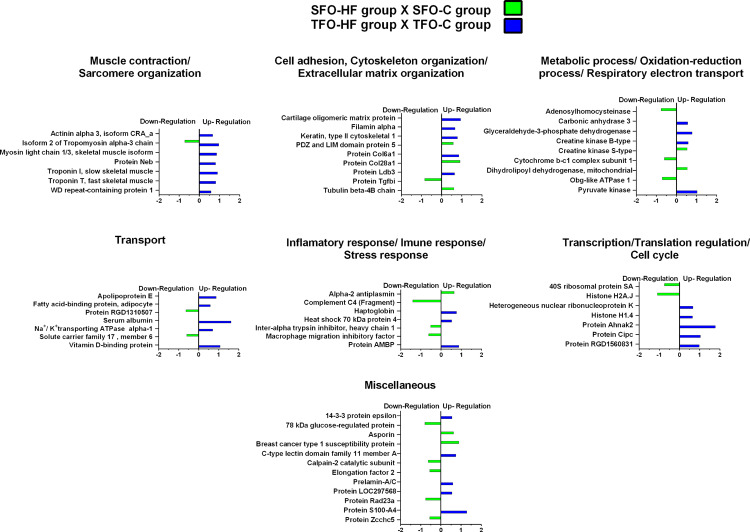
High-fat diet effects on tendon proteome of the offspring. Histogram of protein abundance levels from intergroup analysis considering only proteins with down-regulation and up-regulation (*p* ≤ 0.05), with a ln(fold change) of at least (≥) 0.5. SFO-HF, offspring from sedentary fathers exposed to high-fat diet; SFO-C, offspring from sedentary fathers exposed to control diet; TFO-HF, offspring from trained fathers exposed to a high-fat diet; TFO-C, offspring from trained fathers exposed to control diet. The *X*-axis represents the natural logarithm of the ratio between the treatments (**green: SFO-HF:SFO-C ratio; blue: TFO-HF:TFO-C ratio)**. All altered proteins are grouped according to their biologic process as noted in Gen Ontology (GO). The proteins were considered reliably identified only if presenting an FDR < 1% and at least two matching peptides. Supplementary Data 3 shows detailed information about each protein outlined in this figure.

Supplementary Data 3 shows fold-change, *p*-values of factors (diet and training) and *p*-values of Tukey’s multiple comparisons test related to regulated proteins. The effects of high-fat diet on the offspring’s tendon proteome were grouped according to their biological processes and are presented in Figure 5. Regarding SFO-HF group, the abundance of 25 proteins was shown to be altered when compared with SFO-C (8 protein increased and 17 decreased). In this analysis (SFO-HF: SFO-C), proteins were mainly related to muscle contraction and sarcomere organization (1 downregulated); cell adhesion, cytoskeleton organization/extracellular matrix organization (3 upregulated and 1 downregulated); metabolic process, respiratory electron transport and oxidation-reduction process (2 upregulated and 3 downregulated), transport (2 downregulated); inflammatory response/immune response/stress response (1 upregulated and 3 downregulated); transcription regulation/translation/cell cycle (2 downregulated), and miscellaneous (2 upregulated and 5 downregulated).

Additionally, from TFO-HF group, abundance levels of 34 proteins were shown to be altered when compared with the TFO-C group (34 proteins increased), proteins were mostly related to muscle contraction and sarcomere organization (7 upregulated); cell adhesion, cytoskeleton organization/extracellular matrix organization (5 upregulated); metabolic process, respiratory electron transport and oxidation-reduction process (4 upregulated), transport (5 upregulated); inflammatory response/immune response/stress response (3 upregulated); transcription regulation/translation/cell cycle (5 upregulated), and miscellaneous (5 upregulated).

### Paternal RT Promoted Up-Regulation of Essential Proteins Associated to Muscle Contraction, ECM Organization, Transport, and Transcription in the Offspring Exposed to a High-Fat Diet

**FIGURE 6:**
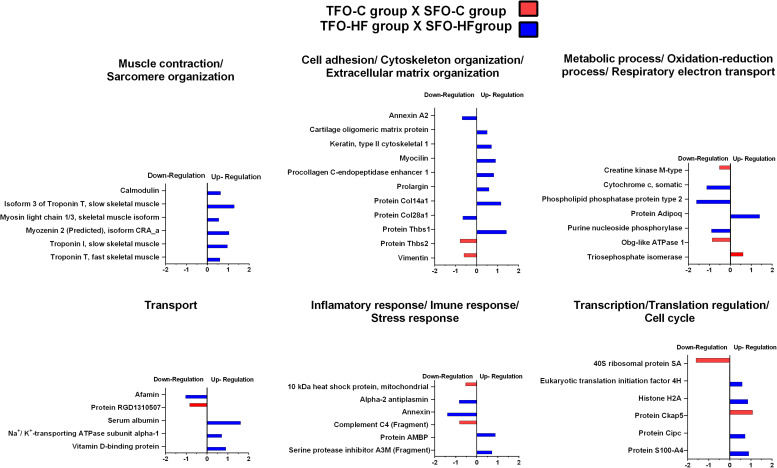
Effects of paternal resistance training on tendon proteome in the offspring exposed to control and high-fat diet. Histogram of protein abundance levels from intergroup analysis considering only proteins with down-regulation and up-regulation (*p* ≤ 0.05), with a ln(fold change) of at least (≥) 0.5. TFO-C, offspring from trained fathers exposed to control diet; SFO-C, offspring from sedentary fathers exposed to control diet; TFO-HF, offspring from trained fathers exposed to a high-fat diet; SFO-HF, offspring from sedentary fathers exposed to high-fat diet; The *X*-axis represents the natural logarithm of the ratio between the treatments **(red: TFO-C:SFO-C ratio, blue: TFO-HF:SFO-HF ratio)**. All altered proteins are grouped according to their biologic process as noted in Gen Ontology (GO). The proteins were considered reliably identified only if presenting an FDR < 1% and at least two matching peptides. Supplementary Data 3 shows detailed information about each protein outlined in this figure.

The effects of paternal resistance training on offspring tendon protein abundance levels exposed to control and high-fat diet were grouped according to their biological processes and are presented in Figure 6 and Table 3. From TFO-C group, abundance levels of 14 proteins were shown to be altered when compared with SFO-C group (3 proteins increased and 11 decreased). In this analysis (TFO-C:SFO-C), proteins were mainly related to cell adhesion, cytoskeleton organization/extracellular matrix organization (2 downregulated); metabolic process, respiratory electron transport and oxidation-reduction process (1 upregulated and 2 downregulated), transport (1 downregulated), inflammatory response/immune response/stress response (2 downregulated), translation (1 upregulated and 1 downregulated), and miscellaneous (1 upregulated and 3 downregulated).

**TABLE 3:** Protein abundance levels related to miscellaneous functions from TFO-HF: SFO-HF analysis considering only proteins with up-regulation and down-regulation (*p* ≤ 0.05) with a delta (Δ) of at least (≥) 0.5-fold change.

Protein description	Primary name	Biological process	*p*-value	Log (e)
				Fold change
TFO-HF:SFO-HF				
Alpha-2-HS-glycoprotein	P24090| FETUA_RAT	Endocytosis	0.0051	0.543057162
Peptidyl-prolyl *cis-trans* isomerase	D3ZZR9| D3ZZR9_RAT	Protein folding	0.0500	1.632129946

Finally, from TFO-HF group, abundance levels of 41 proteins were shown to be altered when compared with the SFO-HF group (25 proteins increased and 8 decreased) (Figure 5 and Table 2). In this analysis (TFO-HF:SFO-HF), proteins were mainly related to muscle contraction and sarcomere organization (7 upregulated); cell adhesion, cytoskeleton organization/extracellular matrix organization (6 upregulated and 2 downregulated); metabolic process, respiratory electron transport and oxidation-reduction process (1 upregulated and 3 downregulated), transport (3 upregulated and 1 downregulated); inflammatory response/immune response/stress response (2 upregulated and 2 downregulated); transcription regulation/translation/cell cycle (4 upregulated) and miscellaneous (2 upregulated).

### Protein–Protein Interactions (PPI)

**FIGURE 7:**
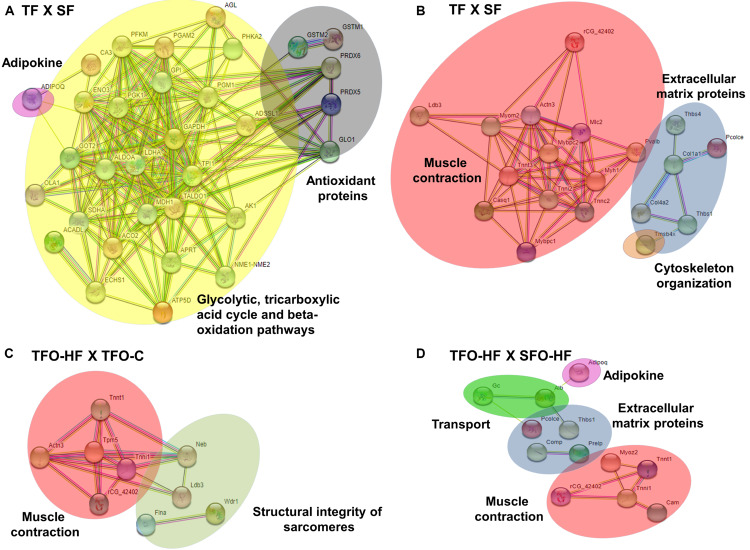
Protein-protein interaction analysis based on STRING network analysis with an interaction confidence score of 0.4. SF, sedentary fathers; TF, trained fathers; TFO-C, offspring from trained fathers exposed to control diet; SFO-HF, offspring from sedentary fathers exposed to high-fat diet; TFO-HF, offspring from trained fathers exposed to a high-fat diet. STRING analysis for differentially abundant proteins in the fathers **(A,B)** and offspring groups **(C,D)**. All altered proteins are grouped according to their biologic process as noted in Gen Ontology (GO). Primary metabolism (yellow), adipokine (pink), oxidation-reduction process (dark gray) and muscle contraction (red), sarcomere organization (light gray), cytoskeleton organization (orange) and extracellular matrix organization (blue), transport (green).

The PPI network of the significantly regulated proteins related to metabolic processes in TF: SF was composed by 33 nodes and 187 edges with an average node degree of 11.13, it indicated high connectivity among oxidative stress protection, glycolysis, citric acid cycle, and fatty acid metabolism proteins (Figure 7A). Upregulated proteins related to sarcomere organization, muscle contraction, ECM organization, cell adhesion, and cytoskeleton organization in TF:SF displayed an interaction network composed of 21 nodes and 58 edges with an average node degree of 5.5. Figure 7B shows the main components of network constructed, such as, troponin T, fast skeletal muscle, actinin alpha 3 and troponin I, type 2, col 1 and col 4.

**FIGURE 8:**
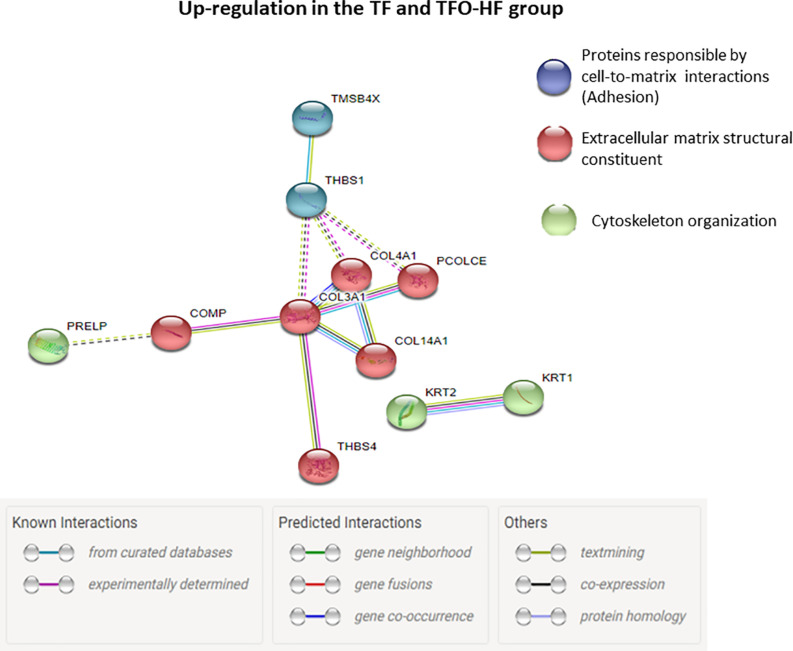
Proteins involved in the same interaction network regarding TF and TFO-HF group. Protein-protein interaction analysis based on STRING network analysis with an interaction confidence score of 0.4. TF, trained fathers; TFO-HF, offspring from trained fathers exposed to a high-fat diet. Blue highlighted nodes are proteins responsible by cell-to-matrix interactions. Red highlighted nodes are extracellular matrix structural constituent. Green highlighted nodes are Proteins responsible by cytoskeleton organization. Upregulated proteins are grouped according to their biologic process as noted in Gen Ontology (GO).

Regarding offspring, the PPI network of the specific upregulated proteins related to sarcomere organization and muscle contraction in TFO-HF:TFO-C groups displayed 12 nodes and 18 edges with an average node degree of 3.00 (Figure 7C). Curiously, a prominent interaction network was found within, myosin light chain 1/3, skeletal muscle isoform, isoform 2 of tropomyosin alpha-3 chain, actinin alpha 3, troponin T, fast skeletal muscle, troponin I, slow skeletal muscle and LIM domain-binding protein. Upregulated proteins related to sarcomere organization, muscle contraction, ECM organization, transport and adipokine in TFO-HF:SFO-HF groups displayed 16 nodes and 11 edges with an average node degree of 1.38 (Figure 7D). No significant protein networks were observed between downregulated proteins, other groups and biological processes. Finally, upregulated proteins related to cell adhesion, extracellular matrix structural and cytoskeleton organization from TF and TFO-HF group were involved in the same interaction network (Figure 8).

## Discussion

The current study was conducted to determine the effects of paternal RT on offspring tendon proteome exposed to different diets. Our results support the initial hypothesis, showing that SFO-HF group presented downregulation of significant proteins related to muscle contraction, ECM organization, transport, immune response, and translation when compared with SFO-C. On the other hand, the effects of paternal RT on tendon proteome were more pronounced when the offspring were exposed to HF diet and most of the regulated proteins presented an increased abundance rather than reduced. Moreover, paternal RT was effective to prevent adiposity gain in the TFO-HF group, representing a phenomenon of major clinical significance. To our knowledge, the present study demonstrated for the first time that paternal exercise modulates the abundance of proteins attributed to the maintenance of tendon functional integrity suggesting that paternal RT can prevent the detrimental effects related to an offspring unhealthy diet. Our findings provide valuable insights into the molecular mechanisms involved in intergenerational inheritance and this approach present innovation in the current knowledge on tendons.

The present proteomic analysis comparing the TF group versus SF group (fathers) demonstrated that 80 proteins were upregulated, while only 1 protein was downregulated. We found that RT increased key antioxidant proteins (i.e., peroxiredoxin 5, lactoylglutathione lyase, glutathione S-transferase mu 2, peroxiredoxin-6 and glutathione S-transferase Mu 1). Cellular reduction-oxidation balance plays a crucial role in the homeostasis of connective tissues such as enthesis and tendon (Bestwick and Maffulli, 2004). Recent evidences suggest that oxidative stress plays a crucial role on tendinopathic changes (Lim et al., 2012; Lee et al., 2017; Fu et al., 2018). Morikawa et al. (2014) reported that the antioxidant enzyme superoxide dismutase 1 deficiency in mice induced several histopathological changes in the supraspinatus tendon enthesis, including decreased type I collagen, formation of misaligned collagen fibers and misaligned 4-layered structure (tendon proper, non-mineralized fibrocartilage, mineralized fibrocartilage and bone) compared with the wild type mice. Taken together, the antioxidant enzymes upregulation in the TF group might play a crucial role in the detoxification of reactive oxygen species following RT, possibly preventing impairments in oxidative capacity. We might speculate that these adaptations may reflect a protection mechanism vital for the maintenance of tendon homeostasis, including appropriate collagen fibers organization.

The RT protocol led to an upregulation of a large number of metabolic proteins (i.e., glycolytic, tricarboxylic acid cycle, and beta-oxidation) in the TF group compared to the SF group, probably due to increased energy demand and the need to maintain the enzymatic operation in the primary metabolism. These proteins present in different metabolic pathway are key enzymes in the conversion of substrates and in ATP production. These changes may contribute to fibroblast survival and ECM turnover in response to RT (Kjaer et al., 2005). Furthermore, the upregulated proteins displayed a protein interaction network with high connectivity (Figure 6). On the other hand, Kaux et al. (2017) demonstrated that proteomic analysis in tendons of rats exposed to eccentric training (treadmill run set at a 15° inclination, 1 h, 3 times per week for 5 weeks) after acute lesions presented a significant decrease in the abundance of glucose metabolism enzymes compared to untrained groups. The discrepancy between these results may be due to differences in intensity, volume, type, and duration of the training protocols employed, as well as acute lesions. Besides, these authors have not addressed the period between last exercise session and euthanasia, which obscure the chronic effects of exercise training on proteomic profile.

Surprisingly, no prominent proteomic adaptations were seen in offspring exposed to control diets whose fathers underwent RT (TFO-C) compared to SFO-C, suggesting limited effects of paternal exercise. Krout et al. (2018) reported that the benefits of paternal exercise on body fat and offspring insulin resistance in skeletal muscle were more noticeably observed when the offspring were exposed to an HF diet. The paternal RT effects on offspring tendon proteomic profile were more evident in the presence of HF diet than in low-fat diet fed to the offspring. The evaluation of only single point in time is a considerable limitation of this study. A more effective strategy would involve displaying protein abundances in different time-points since the beginning of dietary intake in order to clarify the time-course effects of standard diet on offspring tendon proteome.

On the other hand, an important finding of the current study was that SFO-HF demonstrated a decrease of proteins involved in tendon remodeling when compared with SFO-C group. Our results corroborate with transcriptome study which demonstrated that ApoE^–/–^ associated with HF diet, promotes mRNA-level dysregulation of signaling pathways related with ECM synthesis, and repair in the tendon, suggesting mechanism of hypercholesterolemia-induced tendinopathy (Li et al., 2019). TGF-β superfamily orchestrates essential cellular processes that include proliferation, differentiation, and growth of the ECM (Klein et al., 2002). Downregulation of the protein TGFβ1 in present study might indicate a panel of unfavorable remodeling processes inherent to HF diet. This result substantiates the decreased abundance of 40S ribosomal protein in the present study. Ribosomal proteins are responsible for ribosome assembly and protein translation, which is crucial for cell survival (Zhou et al., 2015). Impairment of any of these two cellular processes can severely retard cell growth (Zhou et al., 2015). The SFO-HF displayed a reduced abundance of macrophage migration inhibitory factor (MIF). Although MIF is a proinflammatory cytokine involved in many inflammatory reactions and disorders, recent studies indicated that MIF may be a mediator of anti-inflammatory and immunosuppressive macrophage functions (Yaddanapudi et al., 2013; Peng et al., 2018). Furthermore, the decrease of MIF can inhibit cell proliferation due to decreased expression of cell cycle regulators (Denz et al., 2010) and autophagy suppression (Lee et al., 2016).

Additionally, our data indicated that asporin abundance levels were upregulated in rats exposed to HF diet. Asporin, a class I small leucine-rich proteoglycan, regulates chondrogenesis, and inhibits TGFβ1-induced expression of matrix genes (Nakajima et al., 2007), acting as a critical regulator of joint diseases, including osteoarthritis (Coburn, 2005). Moreover, asporin regulates collagen mineralization, which is a common mechanism of tendinopathy (Xu et al., 2015). Thus, one possibility that should not be ruled out is that asporin overexpression might promote tendon mineralization and possibly contribute to disability and mechanical dysfunctions (Kalamajski et al., 2009; Houari et al., 2014). Further, HF diet promotes downregulation of proteins with multiples metabolic activity functions and responsible by chemical signalizing in primary metabolism (adenosylhomocysteinase cytochrome complex, obg-like ATPase 1, solute carrier family 17 and 78 kDa glucose protein). Reduced abundance of these proteins might compromises tissue microenvironment, metabolism homeostasis and consequently cellular longevity.

Previous data reported detrimental effects of HF diet on tendon biomechanical and morphological properties (Boivin et al., 2013; Grewal et al., 2014). Diet switch from HF diet to low-fat diet seems to resolve metabolic dysfunction, but it was not able to reverse tendinopathic changes (Studentsova et al., 2018). Here, we observed the increase of several proteins related to structural organization of ECM components (cartilage oligomeric matrix protein, filamin alpha, keratin type II cytoskeletal, protein Col6a1) in TFO-HF group when compared with the TFO-C group, indicating that apparently the progression toward a more degenerative phenotype would have a later onset with paternal RT. Despite highly significant, it remains to be determined whether these adaptations at the protein level are enough to attenuate the disorganized collagen and deterioration of tendon biomechanical properties related to HF diet. Moreover, there was no significant reduction of proteins related to tissue damage, repair, ECM structural stability (Figure 5), suggesting that possibly there may be a delay in maladaptive effects inherent to our HF diet rodent model.

Regarding plausible mechanisms, Stanford et al. (2018) described that paternal exercise training significantly regulates multiple classes of small RNAs in sperm, suppressing some harmful paternal dietary effects on small RNA levels, and thus implicating these small RNAs as potential mediators in the transmission of paternal environmental information to the next generation. Considering these findings, and a possible mechanistic explanation, we speculate that offspring proteome would be more influenced when the offspring were exposed to a HF diet. This brief explanation would be a reasonable description to the minimal changes seen in TFO-C compared with SFO-C, and might clarify the relevant proteomic changes seen in TFO-HF compared with SFO-HF.

It is worth noticing that paternal RT promotes a significant increase of proteins related to muscle contraction and sarcomere organization when compared to offspring from sedentary fathers in both high-fat diet (TFO-HF: SFO-HF) and those not exposed to HF diet (TFO-C: SFO-C). According to Subramanian and Schilling (2015), tendon morphogenesis is mediated by interactions with skeletal muscle and bidirectional communication between tissues is essential for tendon integrity maintenance. Moreover, such relationship is potentially accentuated in response to the exercise training (Lidstone et al., 2016). It is reasonable to suspect that cross-talk between tenocytes and muscle cells occur to maintain connectivity and normal tendon remodeling, as well as strengthen attachments under tension (Barin et al., 2019). Possibly, healthy heterotypic interactions between tissues might help mitigate the deleterious effects inherent to HF diet.

It has been widely shown that the tendon structure adapts in response to mechanical loading modifying mainly the ECM architecture and composition (Marqueti et al., 2018). An important outcome of the present study was a considerable increase in abundance of several proteins related to cell adhesion, cytoskeleton organization and ECM organization in the TFO-HF group when compared to the SFO-HF group. Type XIV collagen up-regulation is associated with regulation of assembly and linear fibril growth, which can be important for stabilization of immature fibrils (Ansorge et al., 2009). Moreover, this protein is often present in areas of high mechanical stress, indicating it potentially has a role in maintaining mechanical properties of tissues (Ansorge et al., 2009). Procollagen C-endopeptidase enhancer 1 is a crucial enzyme for accurate and efficient conversion of fibrillar procollagens to their self-assembling monomers (Vadon-Le Goff et al., 2011). Upregulation of this protein might represent an adaptation mechanism to maintain enzymatic operation in the tendon remodeling (Bourhis et al., 2013). At the same time, TFO-HF group presented increased abundance levels of glycoproteins responsible for cell–matrix interactions. The enhancement in the cartilage oligomeric matrix protein and thrombospondin 1 (Thbs1) could facilitate ECM structural support, mechanical stability, and adhesion between cells (Sodersten et al., 2006; Wang et al., 2017). It is worth mentioning that Procollagen C-endopeptidase enhancer 1 and Protein Thbs1 changed in the same direction (i.e., up-regulation) in the two groups involved with exercise training (i.e., TF and TFO-HF group), representing a relevant overlapping between fathers and the offspring. The upregulated proteins in these two groups generated a protein network that showed relevant connectivity (Figure 8). Considering these molecular findings, we suggest that paternal RT can be a relevant intervention capable of inducing significant effects on offspring proteins involved in ECM necessary for structural arrangement and mechanical function.

A novelty of this work was the finding of protein col28a1, increased after HF diet exposure, but paternal HF significantly prevents such increase. The collagen XXVIII is widely detected in the sciatic nerve at the basement membrane of specific Schwann cells surrounding the nerve fibers (Veit et al., 2006). However, the discussion of our findings is not an easy task, because there is no evidence of their specific functions in the tendon (Gebauer et al., 2016). Moreover, RT-PCR revealed a broader expression of collagen XXVIII in newborn than in adult mice (Gebauer et al., 2016), which limits the interpretation of results up to now described.

Another finding from this study was that TFO-HF presented high abundance levels of adiponectin when compared with SFO-HF group. In human clinical studies, adiponectin has shown the ability to improve insulin sensitivity, tenocyte progenitor cells proliferation and differentiation, which makes it a therapeutic agent in treating diabetic tendinopathy (Rothan et al., 2013). Presumably, adiponectin might represent a promising biological target to prevent tendon abnormalities related to HF-diet. In animals models, adiponectin-deficient mice demonstrated a near-normal insulin sensitivity when fed with control diet, however, developed insulin resistance in skeletal muscle after 2 weeks of exposure to HF diet (Maeda et al., 2002). In the same experimental model, the area under the curve of blood glucose did not reveal significant differences between offspring groups (de Sousa Neto et al., 2020), which may be related to Wistar rats not responding pronouncedly to an HF diet when compared to other mouse strains. Future studies will be required to evaluate the link between changes of adiponectin and glucose uptake by tenocytes besides glucose transporters.

**FIGURE 9:**
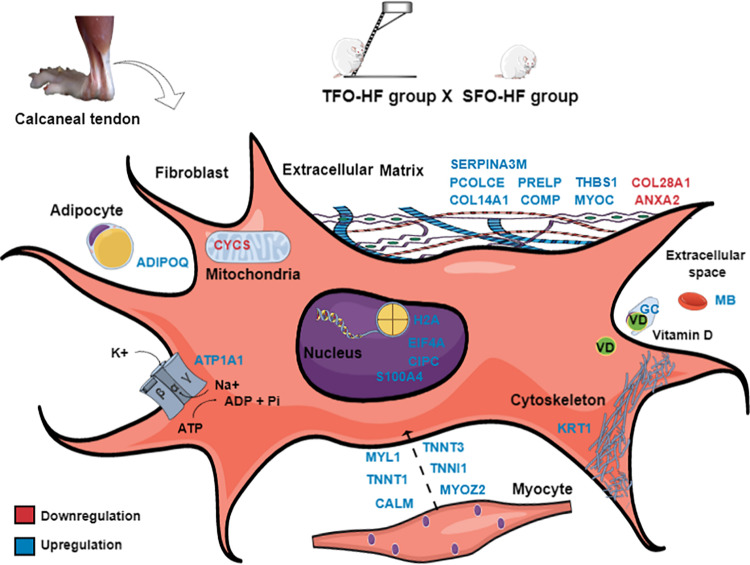
Paternal resistance training affects offspring calcaneal tendon proteome exposed to high-fat diet. SFO-HF, offspring from sedentary fathers exposed to high-fat diet; TFO-HF, offspring from trained fathers exposed to a high-fat diet. The main upregulated (red) and downregulated (blue) proteins in the analysis (TFO-HF: SFO-HF). Adipocyte = Protein Adipoq (ADIPOQ). Cell membrane = Sodium/potassium-transporting ATPase subunit alpha-1 (ATP1A1). Cytoskeleton = Keratin, type II cytoskeletal 1 (KRT1). Extracellular space = Vitamin D-binding protein (GC), Serum albumin (MB). Extracellular matrix = Annexin A2 (ANXA2), Cartilage oligomeric matrix protein (COMP), Myocilin (MYOC), Procollagen C-endopeptidase enhancer 1 (PCOLCE), Prolargin (PRELP), Protein Col14a1 (COL14A1), Protein Col28a1 (COL28A1), Serine protease inhibitor A3M (SERPINA3M), Thrombospondin-1 (THBS1). Mitochondrion = Cytochrome c, somatic (CYCS). Myocyte = Calmodulin (CALM), Isoform 3 of Troponin T, slow skeletal muscle (TNNT3), Myosin light chain 1/3, skeletal muscle isoform (MYL1), Myozenin 2 (MYOZ2), Troponin I, slow skeletal muscle (TNNI1), Troponin T, fast skeletal muscle (TNNT1). Nucleus = Eukaryotic translation initiation factor 4H (EIF4A), Histone H2A (H2A), Protein Cipc (CIPC), Protein S100-A4 (S100A4).

The present study demonstrated an increase of proteins for transporting molecules involved in tendon metabolism and ions bioavailability in TFO-HF group when compared with SFO-HF group. It has been demonstrated that vitamin D binding protein has a role in maintaining the total levels and the amount of free vitamin D (Bikle and Schwartz, 2019). In this context, Angeline et al. (2014) found that decreased levels of vitamin D influence early healing after rotator cuff repair, resulting in decreased fibrocartilage formation and disorganized collagen. The authors suggest that one possible beneficial function of vitamin D in the healing process is to decrease tendon inflammation (Angeline et al., 2014) by downregulating the cellular response to tumor necrosis factor-α, and by regulating matrix metalloproteinase 9 (Bahar-Shany et al., 2010; Dougherty et al., 2016). In the present study, we speculate that the upregulation vitamin D binding protein herein described may help to control the inflammation in order to maintain tendon morphology. Another protein, sodium/potassium-transporting ATPase catalyzes the hydrolysis of ATP coupled with the exchange of sodium and potassium ions across the membrane (Xie and Cai, 2003). An increase of this protein can contribute to create a proper electrochemical gradient, providing the energy for active transport of ions (Xie and Xie, 2005). This regulatory response becomes increasingly important in muscle-tendon junction when it is required a strongest excitation in response to exercise (Gundersen, 2011). A schematic representation of fibroblast was used to clarify the location, the up and downregulation, and the role of the main proteins identified in this study (TFO-HF:SFO-HF) (Figure 9). Proteins that are differentially regulated by paternal RT are particularly promising candidate proteins to transduce exercise-induced tendon health benefits and they represent specific targets for the development of future biomarkers and therapeutics.

To our knowledge, this is the first study that used the RT modality in fathers and we elucidated the importance of this type of training in future generations. This study contributes to better understanding of tendon biology and clarifies more profoundly the molecular networks behind physiological adaptations promoted by paternal RT. Our results open new perspectives for studies based on transcriptomics, metabolomics, and functional assays. Despite all the proteomic profiles, the lack of other analyses, such as morphological properties, tendon functional assessments would be relevant to clarify adjacent mechanisms involved in intergenerational inheritance. The size of the tendon was small (∼80 μg of tissue weight) and only allowed us to obtain an adequate amount of proteins for proteomic analysis. Due to the specific procedures performed we cannot use the processed sample to other analyzes that require different methods of extraction. Modifications in sperm provide a potential molecular basis to explain the contribution of the father’s lifestyle on the offspring phenotypes and non-coding RNAs are probabilistic epigenetic mechanisms. Further investigations are required to evaluate the link between sperm epigenetic status and tendon proteome.

## Conclusion

Paternal RT affect the abundance of numerous proteins in the tendon and HF diet results in disturbance of important proteins which might explain tendon disorders associated to an unhealthy diet. Most interestingly, paternal RT modulates pathways in the tendon of the offspring, being such modulation more evident when the offspring is subjected to HF diet. Most of the modulated proteins are associated to biological pathways related to tendon protection and damage recovery, such as ECM organization, transport and inflammatory mediators, suggesting a protective effect of paternal exercise against potential harmful effects of a HF diet. Tendency to tendon diseases and higher prevalence of tendon injuries in the offspring could be partially explained by the father behavior.

## Data Availability Statement

The spectra were saved as raw files for further analyses and were deposited at the Proteome Xchange (Perez-Riverol et al., 2015) consortium via the Mass Spectrometry Interactive Virtual Environment (MassIVE) platform (Jarnuczak and Vizcaíno, 2017) under the ID: PXD016182 and MSV000084545.

## Ethics Statement

The animal study was reviewed and approved by Ethics Committee on Animal Experimentation from the Catholic University of Brasilia (Protocol No. 010/13).

## Author Contributions

IS, RT, JD, OF, JP, and RC conceived and planned the design of the experiments. IS, RT, LS, EL, GP, and JA performed the experiments and analyzed the data. MS, CR, HD, MC, and WF performed the methods of label-free quantification and bioinformatics. IS, RT, JP, OF, JD, WF, and RC interpreted the results and worked in the writing of manuscript. OF, JD, AA, WF, and RC involved in planning and supervised the work, also contributed to the design and implementation of the research and provided critical feedback and analysis of the manuscript. All authors discussed the results and contributed to the final manuscript.

## Conflict of Interest

The authors declare that the research was conducted in the absence of any commercial or financial relationships that could be construed as a potential conflict of interest.

## Footnotes

1 http://www.metaboanalyst.ca/

## Supplementary Material

FIGURE S1 Classification of the identified proteins according to the GO terms: cellular localization **(A)**, biologic process **(B)**, and molecular function **(C)**.

FIGURE S2 Venn diagram representation of the identified proteins by Nano LC-MS analysis in the fathers **(A)** and offspring groups **(B)**, showing high consistency of protein identification among the groups. Proteins identified in non-overlapping groups were classified by their biological process.
