# Supplementary figures and images for: Paternal Resistance Training Modulates Calcaneal Tendon Proteome in the Offspring Exposed to High-Fat Diet
Source: Front Cell Dev Biol. 2020 Jun 16;8:380. doi: 10.3389/fcell.2020.00380 (PMC7325979; doi:10.3389/fcell.2020.00380)

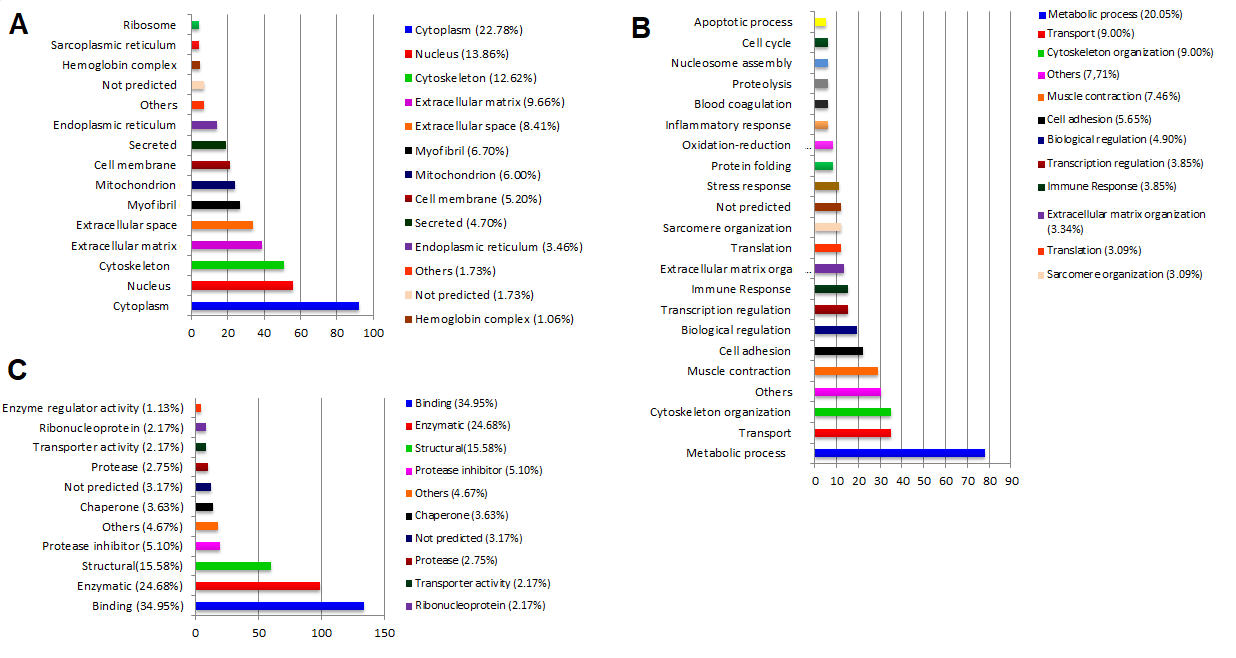

Supplement: FIGURE S1 — Classification of the identified proteins according to the GO terms: cellular localization (A), biologic process (B), and molecular function (C). [file Image_1.tif]

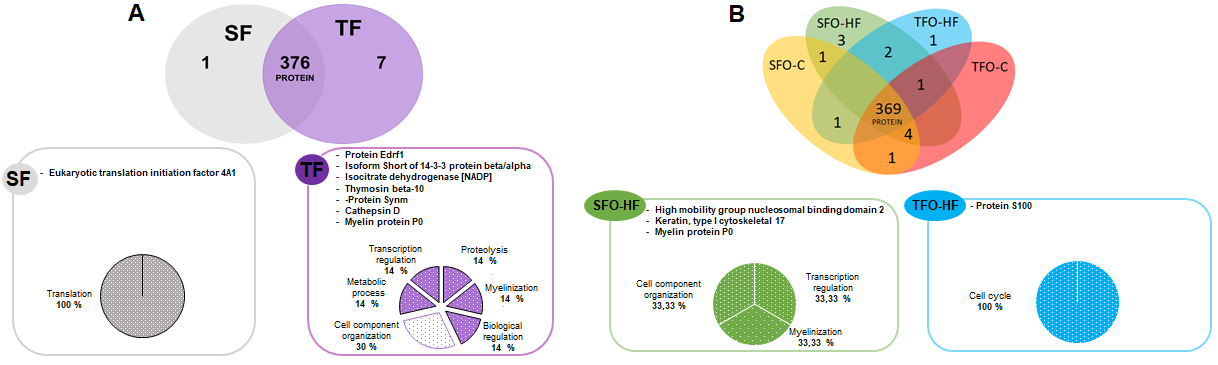

Supplement: FIGURE S2 — Venn diagram representation of the identified proteins by Nano LC-MS analysis in the fathers (A) and offspring groups (B), showing high consistency of protein identification among the groups. Proteins identified in non-overlapping groups were classified by their biological process. [file Image_2.tif]
