# Supplementary material for: Paternal Resistance Training Modulates Calcaneal Tendon Proteome in the Offspring Exposed to High-Fat Diet
Source: Front Cell Dev Biol. 2020 Jun 16;8:380. doi: 10.3389/fcell.2020.00380 (PMC7325979; doi:10.3389/fcell.2020.00380)
Supplement: Supplementary file 3 [file Data_Sheet_1.docx]

**Supplementary data 1**

**Sample Power Analysis**

Statistical Power of the Manuscript **Paternal resistance training modulates calcaneal tendon proteome in the offspring exposed to high-fat diet** followed the instruction by Beck (2013)[^1^](#_ENREF_1) and Faul et al (2007)[^2^](#_ENREF_2).

**F tests -** ANOVA: Fixed effects, omnibus, (four groups)

Our research investigated the the effect of paternal resistance training before conception on the tendon proteome of the offspring. Thus, which ones were the parameters used to make the calculations including the desired power and magnitude of difference aiming for protein abundance levels ? Refer to figure 1 as you go through the following steps. The G*Power 3.1.9.4 was used to calculate statistical power analysis.

**Step 1:** We selected “F tests” in the “Test family” panel.

**Step 2:** In the “Statistical test” panel, select “**F tests -** ANOVA: Fixed effects, omnibus, (four groups).”

**Step 3:** In the “Type of power analysis” panel, select “Post hoc: Compute achieved power – given α, sample size, and effect size.”

**Step 4:** In the “Input Parameters” panel, select “Determine” and this will open-up a new panel that allows the user to calculate the effect size for the protein abundance levels. For example (Cartilage oligomeric matrix protein), using the mean and standard deviation of the Table 1 for four groups we can calculate the power of the present study. Such choice is justified because it represents the main dependent variable.

| **TABLE 1. protein abundance levels for Cartilage oligomeric matrix protein** | | |
| --- | --- | --- |
| OFFSPRING GROUPS | MÉDIA | DP |
| SFO-C | 35183 | 17304 |
| TFO-C | 28435 | 18085 |
| SFO-HF | 77349 | 62923 |
| TFO-HF | 250196 | 160451 |

**Step 5**. The “Effect size d²” can be estimated from the mean and standard deviation highlighted (*Table 1*). To estimate the “Effect size d²” using this method, select “Determine” in the “Input Parameters” panel. Next, select “Calculate,” and “Calculate and transfer to main window.” This will transfer the estimated “Effect size f²” of 3.66 (**Figure 2**) to the main window. Select 0.05 for the “(1-α err prob), 6 rats for sample size group 1 and 6 rats for sample size group 2. Select “Calculate,” and observe the “Total sample size” of 6 rats required to achieve a power level of 0.87 (**Figure 2**).

**Figure 2.**


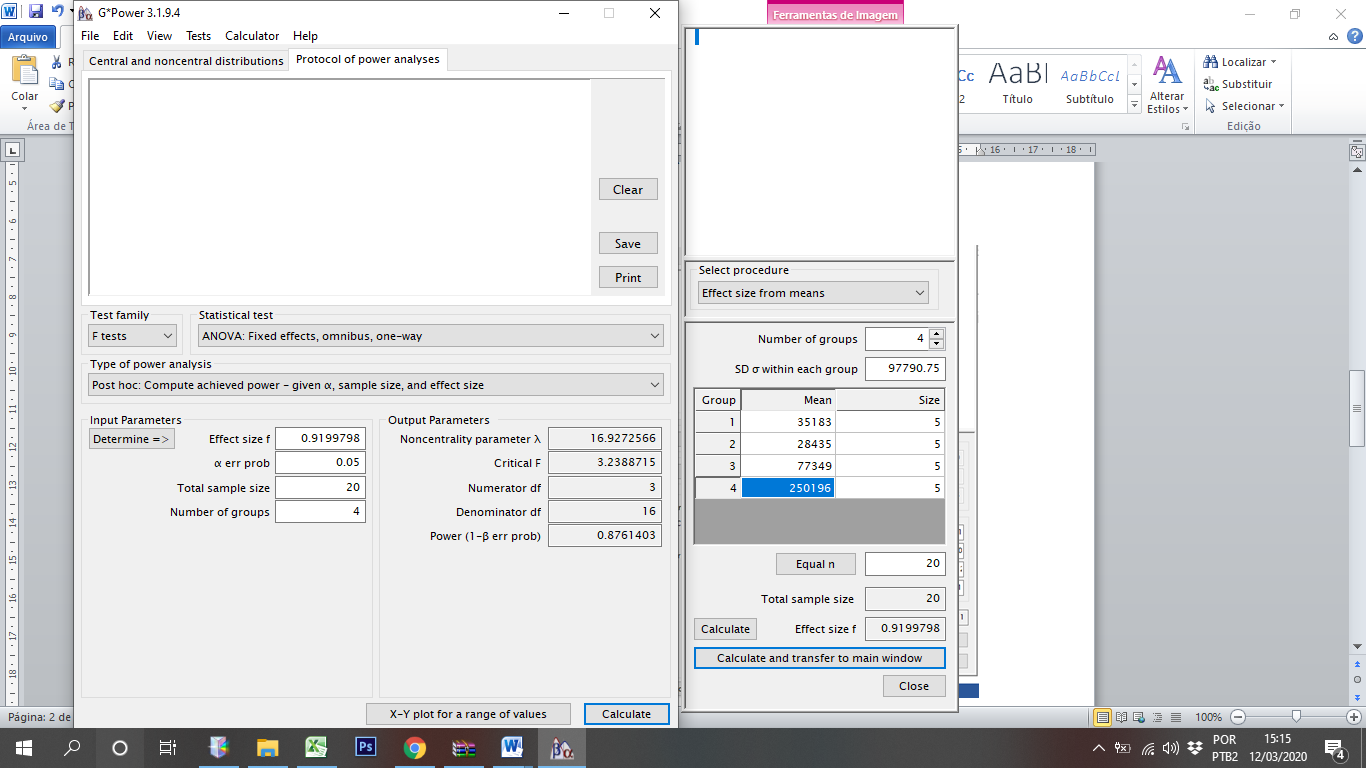


**GPower Protocol**

**F tests -** ANOVA: Fixed effects, omnibus, one-way

**Analysis:** Post hoc: Compute achieved power

**Input:** Effect size f = 0.9199798

α err prob = 0.05

Total sample size = 20

Number of groups = 4

**Output:** Noncentrality parameter λ = 16.9272566

Critical F = 3.2388715

Numerator df = 3

Denominator df = 16

Power (1-β err prob) = 0.8761403

**Table**. Post hoc power analysis of main proteins

|  | **Noncentrality parameter λ** | **Critical F** | **Numerator df** | **Denominator df** | **Power (1-β err prob)** |
| --- | --- | --- | --- | --- | --- |
| **Protein name** |  |  |  |  |  |
| Cartilage oligomeric matrix protein | 16.9272566 | 3.2388715 | 3 | 16 | 0.8761403 |
| Procollagen C-endopeptidase enhancer 1 | 70.1025445 | 3.2388715 | 3 | 16 | 0.9999989 |
| Protein Adipoq | 14.3156059 | 3.2388715 | 3 | 16 | 0.8109712 |
| Protein Col14a1 | 14.0989427 | 3.2388715 | 3 | 16 | 0.8044671 |
| Protein Col28a1 | 16.9894786 | 3.2388715 | 3 | 16 | 0.8774206 |
| Tgf-βi | 15.5936069 | 3.2388715 | 3 | 16 | 0.8457795 |
| Thrombospondin-1 | 20.4595418 | 3.2388715 | 3 | 16 | 0.9327571 |
| Thrombospondin-4 | 153.7635 | 3.2388715 | 3 | 16 | 1.0000000 |
| Vitamin D-binding protein | 20.4595418 | 3.2388715 | 3 | 16 | 0.9327571 |
| 40S ribosomal protein | 15.8394796 | 3.2388715 | 3 | 16 | 0.8518128 |

**References**

**1.** Beck TW. The importance of a priori sample size estimation in strength and conditioning research. J Strength Cond Res. Aug 2013;27(8):2323-2337.

**2.** Faul F, Erdfelder E, Lang AG, Buchner A. G*Power 3: a flexible statistical power analysis program for the social, behavioral, and biomedical sciences. Behav Res Methods. May 2007;39(2):175-191.
